# Development of a pediatric obstructive sleep apnea triage algorithm

**DOI:** 10.1186/s40463-021-00528-8

**Published:** 2021-07-15

**Authors:** D. S. Heath, H. El-Hakim, Y. Al-Rahji, E. Eksteen, T. C. Uwiera, A. Isaac, M. Castro-Codesal, C. Gerdung, J. Maclean, P. J. Mandhane

**Affiliations:** 1 Department of Educational Psychology, University of Alberta, Edmonton, Alberta Canada; 2 Division of Otolaryngology Head and Neck Surgery, Department of Surgery and Pediatrics, University of Alberta, Edmonton, Alberta Canada; 3 Stollery Children’s Hospital, University of Alberta, Edmonton, Alberta Canada; 4 Department of Pediatrics, University of Alberta, Edmonton, Alberta Canada

**Keywords:** Sleep-related breathing disorder, Obstructive sleep apnea, Snoring, Tonsillectomy, Adenoidectomy, Oximetry

## Abstract

**Introduction:**

Diagnosis and treatment of obstructive sleep apnea (OSA) in children is often delayed due to the high prevalence and limited physician and sleep testing resources. As a result, children may be referred to multiple specialties, such as pediatric sleep medicine and pediatric otolaryngology, resulting in long waitlists.

**Method:**

We used data from our pediatric OSA clinic to identify predictors of tonsillectomy and/or adenoidectomy (AT). Before being seen in the clinic, parents completed the Pediatric Sleep Questionnaire (PSQ) and screening questionnaires for restless leg syndrome (RLS), nasal rhinitis, and gastroesophageal reflux disease (GERD). Tonsil size data were obtained from patient charts and graded using the Brodsky-five grade scale. Children completed an overnight oximetry study before being seen in the clinic, and a McGill oximetry score (MOS) was assigned based on the number and depth of oxygen desaturations. Logistic regression, controlling for otolaryngology physician, was used to identify significant predictors of AT. Three triage algorithms were subsequently generated based on the univariate and multivariate results to predict AT.

**Results:**

From the OSA cohort, there were 469 eligible children (47% female, mean age = 8.19 years, *SD* = 3.59), with 89% of children reported snoring. Significant predictors of AT in univariate analysis included tonsil size and four PSQ questions, (1) struggles to breathe at night, (2) apneas, (3) daytime mouth breathing, and (4) AM dry mouth. The first triage algorithm, only using the four PSQ questions, had an odds ratio (OR) of 4.02 for predicting AT (sensitivity = 0.28, specificity = 0.91). Using only tonsil size, the second algorithm had an OR to predict AT of 9.11 (sensitivity = 0.72, specificity = 0.78). The third algorithm, where MOS was used to stratify risk for AT among those children with 2+ tonsils, had the same OR, sensitivity, and specificity as the tonsil-only algorithm.

**Conclusion:**

Tonsil size was the strongest predictor of AT, while oximetry helped stratify individual risk for AT. We recommend that referral letters for snoring children include graded tonsil size to aid in the triage based on our findings. Children with 2+ tonsil sizes should be triaged to otolaryngology, while the remainder should be referred to a pediatric sleep specialist.

**Graphical abstract:**

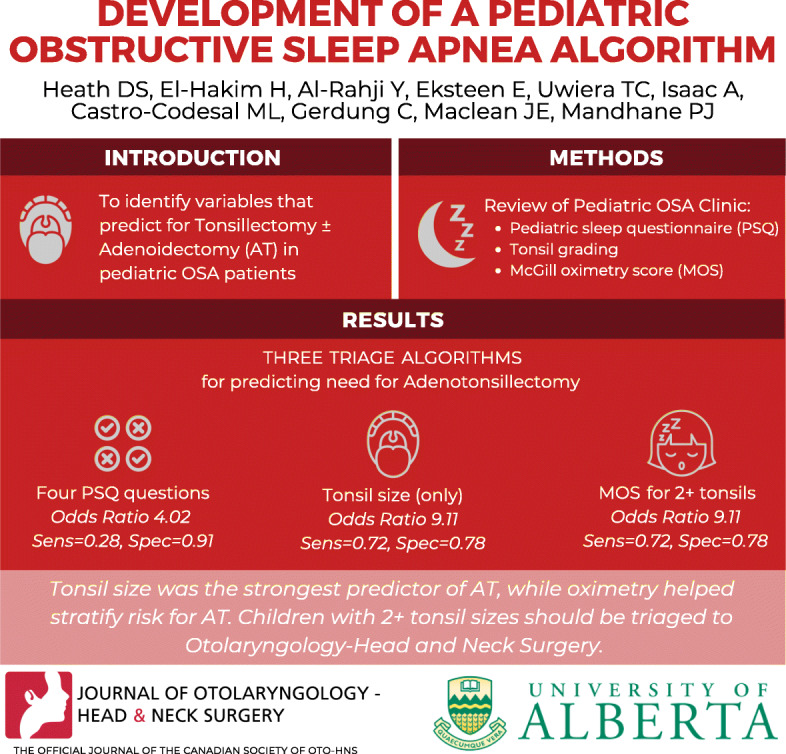

**Supplementary Information:**

The online version contains supplementary material available at 10.1186/s40463-021-00528-8.

## Introduction

Sleep-disordered breathing describes a set of ventilatory disorders ranging from snoring to obstructive sleep apnea (OSA; 1). Prolonged OSA symptoms have been associated with learning difficulties, emotional dysregulation, and behavioural concerns, as well as cardiovascular and metabolic complications [1–3]. Reducing the time between initial investigation and intervention may help prevent these adverse outcomes [4]. An earlier age of onset, and longer duration of sleep-related breathing disorder (SRBD) symptoms is associated with greater behavioral problems, emphasizing the need for earlier identification and treatment [5].

Children may present with OSA due to enlarged adenoids and tonsils, craniofacial abnormalities, obesity, abnormal muscle tone of the upper airway, and abnormal drive to breathe [1]. As a result, the multiple causes of childhood OSA make it challenging to determine which specialty should first see the OSA patient. The most common cause of childhood OSA is adenotonsillar hypertrophy. In addition to adenotonsillar hypertrophy, children who snore for greater than 3 months are more likely to be male, experience obesity, and are breastfed [6]. As such, tonsillectomy and/or adenoidectomy (AT) by an otolaryngologist is the most common treatment for OSA [7, 8]. Children with OSA may also present with associated comorbidities such as allergic and non-allergic rhinitis, restless leg syndrome (RLS [9];) and gastroesophageal reflux (GERD [10];). As a result of these multiple overlapping causes of OSA, physicians with specialty training in respirology may also see and treat OSA patients. Otolaryngology – head and neck surgery residents, while knowledgeable on OSA, had variable confidence in managing OSA surgeries [11] highlighting the complexity and importance of effective triaging and referral protocols for OSA patients. Further, wait-times for patients can be reduced by identifying which specialty the OSA patient should be referred to first [1, 12–14].

Polysomnography (PSG) is the golden standard for diagnosing OSA in children [7]. Unfortunately, PSG is a time-consuming and expensive test with limited resources in pediatric centers worldwide [13]. Overnight oximetry studies are often used to screen for OSA [7, 15, 16], although they have limited sensitivity. Both PSG and overnight oximetry do not determine the cause of an individual’s OSA. While not diagnostic for OSA, tonsil size and adenoid size may help determine which children are AT surgical candidates [17]. We used data from our pediatric OSA clinic to identify predictors of AT, including tonsil and adenoid size and overnight oximetry [12]. We subsequently developed an algorithm for triaging patients to either pediatric respirology or otolaryngology.

## Method

### Study population

Children seen at the pediatric OSA clinic at the Stollery Children’s Hospital in Edmonton, Alberta, Canada, between August 2012 and May 2020, who consented to have their charts reviewed for research purposes, were included in this analysis [12]. Children with comorbidities were excluded at triage. Details of the processes and questionnaires associated with the pediatric OSA clinic have been described previously [12]. Briefly, parents completed questionnaires and some testing, such as overnight oximetry, before being seen in the clinic. For clinical care purposes, clinic data collection was approved by the Alberta Office of Information and Privacy (004604). This analysis was approved by the University of Alberta ethics board (Pro00089369).

### Study variables

#### AT (primary outcome variable)

AT history was obtained from the patient’s chart after their clinic visits or was parent-reported through our medical intake questionnaire.

#### Tonsil size (primary predictor variable)

Tonsil size data, obtained from patient charts, was graded using the Brodsky five-grade scale [17].

#### McGill oximetry score (MOS; secondary predictor variable)

Children completed an overnight oximetry study before being seen in the clinic (i.e., no treatment was started by the clinic team). A McGill oximetry score (MOS) was assigned to each study based on the number and depth of oxygen desaturations [15].

#### Additional predictor variables assessed by questionnaires

Before being seen in the clinic, parents were asked to complete the Pediatric Sleep Questionnaire (PSQ [18];) and screening questionnaires for Restless Leg Syndrome (RLS), Gastroesophageal Reflux Disease (GERD) and the (Nasal Obstruction Symptom Evaluation (NOSE) survey [19];). Participants were considered to be at risk for RLS if they endorsed at least one RLS symptom. Children with two or more GERD symptoms were considered to have GERD. Children with a NOSE score of 10 or greater were considered to have significant rhinitis symptoms [19].

### Statistical analyses

Univariate and multivariate logistic regression analyses (controlling for otolaryngology physicians) were completed to identify predictors of AT (outcome variable). Three triage algorithms were generated based on the univariate and multivariate results to predict AT. The *p*-value was set at *p* < 0.05. Statistical analyses were performed using Stata/MP 15.1 (Stata Corporation).

## Results

**Fig. 1:**
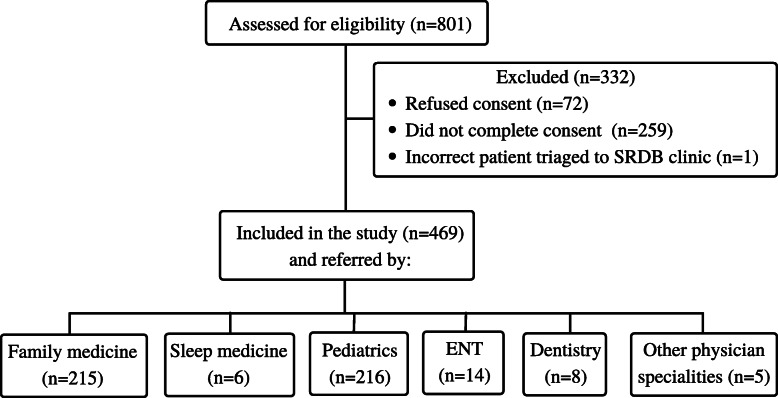
Figure one depicts the distribution of participants from the OSA clinic

There were 469/801 patients included in this analysis as 259 children did not have a research consent (Fig. 1), one patient was incorrectly triaged to our clinic [1], and 72 refused consent for research. Of the 469 children included in the analyses, 46% (215/469) were referred from family medicine, 46% from pediatrics (216/469), with the remainder, referred from dentistry, otolaryngology, and other physician specialties [5]. There were 89% (418/469; Table 1) of children who reported ever snoring, and 65% (306/469) snored over half the night. The mean age at the time of the clinic visit was 8.2 years (*SD* = 3.6), and 207/436 (47%) of participants were female.

**Table 1:** Demographic characteristics and results

Categorical variables	Received AT (n)	No AT (n)	***P***-value	Univariate OR (95% CI)	Adjusted OR (95% CI) ^**a**^
Female	55.2% (79/143)	42.7% (128/293)	0.04	1.50 (1.03–2.20)	1.71 (1.05–2.80)
Family income > $60,000	91.9% (124/135)	94.3% (265/281)	0.14	0.14 (0.28–1.20)	0.23 (0.80–0.69)
Ever snore? Yes	93.7% (134/143)	87.0% (261/300)	0.05	1.95 (0.99–3.85)	1.13 (0.47–2.69)
Struggle to breathe while asleep? Yes	56.0% (80/143)	37.7% (113/300)	0.00	2.53 (1.61–3.99)	2.11 (1.19–3.73)
Witnessed apneas? Yes	46.2% (66/143)	27% (81/300)	0.00	3.23 (1.94–5.36)	2.40 (1.27–4.52)
Mouth breathes during the day? Yes	74.8% (107/143)	56% (169/300)	0.00	2.24 (1.36–3.69)	2.20 (1.16–4.16)
Dry mouth in the morning? Yes	62.2% (89/143)	48% (144/300)	0.01	2.50 (1.40–4.48)	1.90 (0.92–3.92)
Tonsil Score 3	16.8% (24/143)	14.7% (44/300)	0.00	3.41 (1.57–7.39)	2.55 (1.01–6.45)
Tonsil Score 4	26.6% (38/143)	9% (27/300)	0.00	7.96 (3.63–17.45)	5.71 (2.19–14.87)
At least one parent reported RLS symptom	85.3% (122/143)	82.3% (247/300)	0.53	1.18 (0.71–1.97)	1.25 (0.65–2.41)
At least two parent-reported GERD symptoms	19.6% (28/143)	26.3% (79/300)	0.01	1.77 (1.11–2.83) 1.53 (0.84–2.78)	
NOSE score of 10 or greater	81.8% (117/143)	67% (201/300)	0.01		
Tonsil size (Grade 0)	0.02% (3/143)	0% (0/300)			
Tonsil size (Grade 1)	7.0% (10/143)	16.7% (50/300)	0.85	0.94 (0.47–1.86)	0.28 (0.11–0.62)
Tonsil size (Grade 2)	26.6% (38/143)	14% (42/300)	0.00	3.6 (2.12–6.12)	1.17 (0.56–2.42)
Tonsil size (Grade 3 and 4)	40.6% (58/143)	9.3% (28/300)	0.00	7.84 (4.54–13.54)	2.37 (1.17–4.82)
McGill oximetry score (2+)	12.6% (18/143)	4% (12/300)	0.001	3.42 (1.62–7.21)	1.34 (0.57–3.15)
Continuous variables					
**Categorical variables**	**Received AT**	**No AT**	**P-value**	**Univariate OR (95% CI)**	**Adjusted OR (95% CI)** ^**a**^
Age in years (SD, range)	7.5 (3.4, 1.5–16.9)	8.5 (3.6, 0.89–17.8)	0.73	0.94 (0.89–1.00)	0.99 (0.91–1.06)

^a^Adjusted for ENT surgeon

### Univariate results

Children with tonsil sizes graded as 3 were 3.41 times more likely to have a AT compared to children with tonsils graded 1 (95% CI 1.57, 7.39; *p* < 0.05; Table 1) while children with tonsil sizes graded as 4 were 7.96 times more likely to have a AT compared to children with tonsils graded as 1 (95% CI 3.63, 17.45; p < 0.05). Children with three or more positive PSQ questions were significantly more likely to receive a AT. Four PSQ questions were significantly associated with a child having a AT: (1) struggles to breathe at night; (2) witnessed apneas; (3) mouth breathes during the day; and (4) has a dry mouth in the morning. Children with a MOS of 2 or greater were 3.42 times more likely to receive a T & A than those with a MOS less than 2 (95% CI 1.62, 7.21; *p* < 0.05).

### Multivariate predictors of AT

Tonsil size was the only predictor of AT when controlling for ENT surgeons.

### Triage algorithms

Three triage algorithms were developed based on the univariate and multivariate results. Algorithms were based on 1) four PSQ questions, 2) tonsil size and 3) tonsil size and oximetry. A model based on oximetry alone is available in the supplementary material. No children with a prior AT (*n* = 18) required a second AT intervention (0% AT) over our study timeframe. Therefore, children with a prior AT are always initially triaged to respirology in all three algorithms.

#### PSQ based algorithm (Fig. 2)

Children with four of four PSQ questions positive (identified from the univariate analysis; *n* = 66) were given a 'Tonsil Score' of four out of four, and were triaged to ENT (56% AT). Subsequently, children with at least one RLS symptom (*n* = 318) were triaged to respirology (25% AT). Children with two or more GERD symptoms or a NOSE score greater than or equal to 10 were also triaged to respirology (32% AT). Out of the remaining 34 children, children with three of four PSQ questions positive were given a 'Tonsil Score' of three out of four and were triaged to ENT (n = 3; 50% AT). The remaining 31 children were triaged to respirology (10% AT). The sensitivity for this model was 0.28, with a specificity of 0.91 (Table 2).

**Fig. 2:**
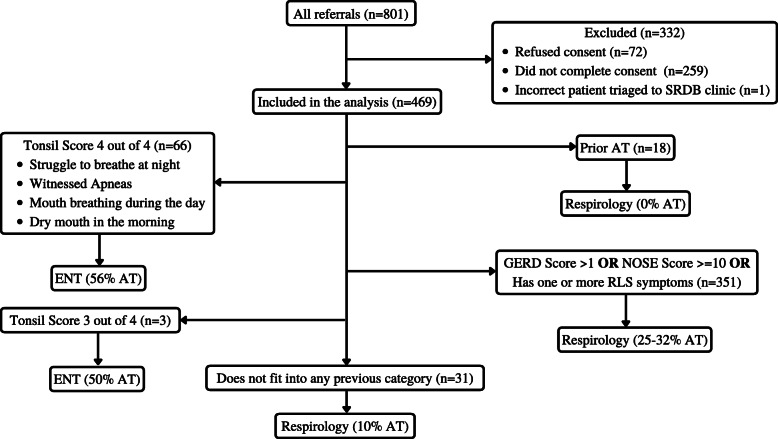
Figure two represents the triage model for children with suspected OSA and PSQ data

**Table 2:** Model metrics

	PSQ Only	Tonsils only	Tonsils + Oximetry
Sensitivity	0.28	0.72	0.72
Specificity	0.91	0.78	0.78
PPV	0.56	0.56	0.56
NPV	0.76	0.88	0.88
False positive rate	0.09	0.22	0.22
True negative rate	0.91	0.78	0.78
True positive rate	0.28	0.72	0.72
False negative rate	0.72	0.28	0.28
Positive Likelihood Ratio	3.18	3.27	3.27
Negative Likelihood Ratio	0.79	0.36	0.36
Odds Ratio	4.03	9.11	9.11

#### Tonsil size-based algorithm (Fig. 3)

Children with graded tonsils 0 or 1 were triaged to respirology (*n* = 63; 17% AT), while those with graded tonsils 2, 3, and 4 were triaged to ENT (*n* = 167; 56% AT). All remaining children were triaged to respirology. The sensitivity of this model was 0.72, and the specificity was 0.78.

**Fig. 3:**
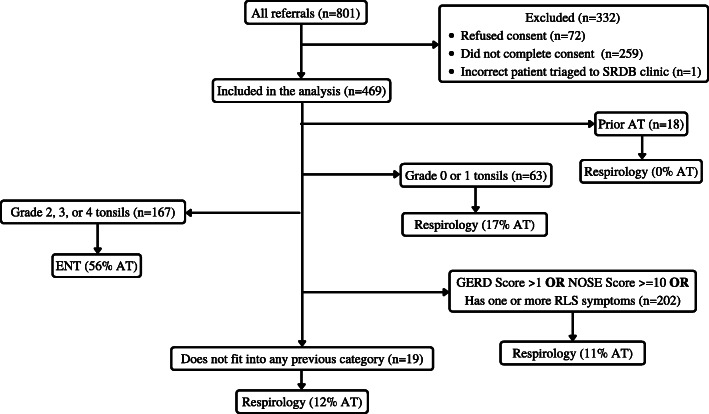
Figure three represents the triage model for children with suspected OSA and tonsil size data

#### Tonsil and oximetry-based algorithm (Fig. 4)

Oximetry did not impact which specialty children were triaged to. However, MOS was associated with AT rates. Among children with graded tonsils 2–4, those with a MOS score of 2–4 had a 78% AT rate (*n* = 21). Children with graded tonsils 2–4 and a MOS score of less than 2 had a 50% chance of a AT (*n* = 122). Children with graded tonsils 2–4 and no oximetry data (*n* = 24) had a 71% AT rate. The metrics for this model were identical to the metrics found in the model without oximetry data.

**Fig. 4:**
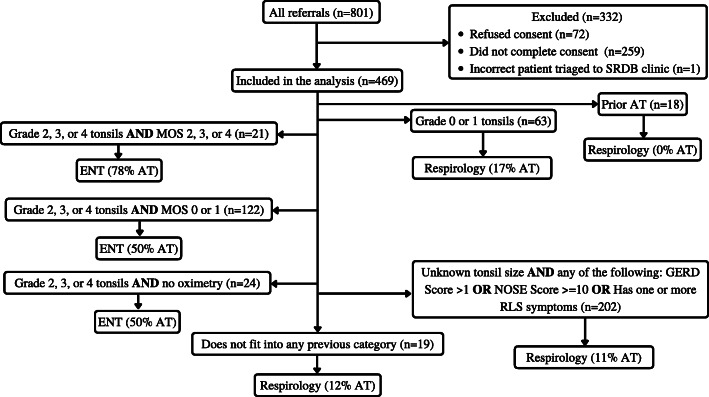
Figure four represents the triage model for children with suspected OSA with both tonsil size and oximetry data

## Discussion

Using data from our pediatric OSA clinic, we found that tonsil size was the strongest predictor of AT in otherwise healthy children. The MOS was useful for stratifying otherwise healthy children with OSA by risk for AT. The triage algorithms generated using this data had a high positive likelihood ratio for identifying children who will have a AT. Based on our findings, we recommend that physicians referring otherwise healthy children with OSA symptoms should i) Provide graded tonsil size in the referral letter and ii) Consider tonsil size when deciding whether to direct refer to ENT or respirology.

Tonsil size has been previously associated with AT despite previous research showing OSA severity was not linearly associated with tonsils size above grade 2 [20]. Consistent with this finding, our triage algorithm recommended referring children with OSA and tonsils grades 2–4 to ENT as they are more likely to be offered AT. As per the original MOS publication, we found that oximetry, and by extension MOS, simply stratified risk for AT rather than identify which specialty a patient should be referred to [15]. However, it was not the scope of this study to predict the improvement of OSA symptoms after AT. Surprisingly, the PSQ, a validated tool to identify children with OSA, did not help triage patients. The PSQ may not help triage patients due to the subjective nature of the PSQ, resulting in parental-report bias and the referral bias of the clinic sample.

The strength in these analyses come from the comprehensive data collected from a community sample of OSA cases. Limitations exist due to the subjective nature of parent-reported symptoms on the PSQ and incomplete data on whether patients received an adenoidectomy and/or tonsillectomy alone. Applying this algorithm to centers with different clinical practices or more complex pediatric populations, such as obese children or those with craniofacial abnormalities, are potential limitations. Additionally, nearly all participants snored, representing a pre-selected sample of children at higher risk for OSA.

Future work would include validating our triage algorithms in a separate clinic sample and follow-up to determine whether OSA symptoms and sleep concerns were resolved following AT. Modifying the algorithm with information from PSG or home sleep testing rather than oximetry alone may further improve the algorithms predictive capacity. Follow-up studies may explore long-term outcomes and comparing post-operative complications [21] for patients who were triaged based on the algorithm versus a control group.

## Conclusion

We aimed to identify an efficient method of triaging pediatric cases of OSA to respirology or ENT. We created three algorithms to predict AT, all of which had similar positive likelihood ratios but different odds ratios. Our results highlight the importance of utilizing the PSQ to guide the referral process. The PSQ, a cost-effective, fast, reliable and valid measure of sleep-related issues [18], may be implemented in primary care to help alleviate long waitlists for sleep studies and initial specialist consultation. Unsurprisingly, tonsil size was the strongest predictor of AT, while oximetry MOS only stratified risk for AT. Based on our results, we recommend that pediatric OSA referral letters include graded tonsil size to aid in the triaging of suspected OSA cases.

## Supplementary Information

**Additional file 1:** Contains an additional triage model that uses oximetry data alone.

## Data Availability

All data generated or analyzed during the present study are included in this published article.

## Abbreviations

AT: Adenoidectomy/Tonsillectomy
ENT: Ear, Nose, Throat
GERD: Gastroesophageal reflux
MOS: McGill Oximetry Score
NOSE: Nasal Obstruction Symptom Evaluation
OSA: Obstructive Sleep Apnea
PSG: Polysomnography
PSQ: Pediatric Sleep Questionnaire
RLS: Restless Leg Syndrome
SRDB: Sleep Related Disordered Breathing
