# Supplementary figures and images for: Development of a pediatric obstructive sleep apnea triage algorithm
Source: J Otolaryngol Head Neck Surg. 2021 Jul 15;50:48. doi: 10.1186/s40463-021-00528-8 (PMC8281470; doi:10.1186/s40463-021-00528-8)

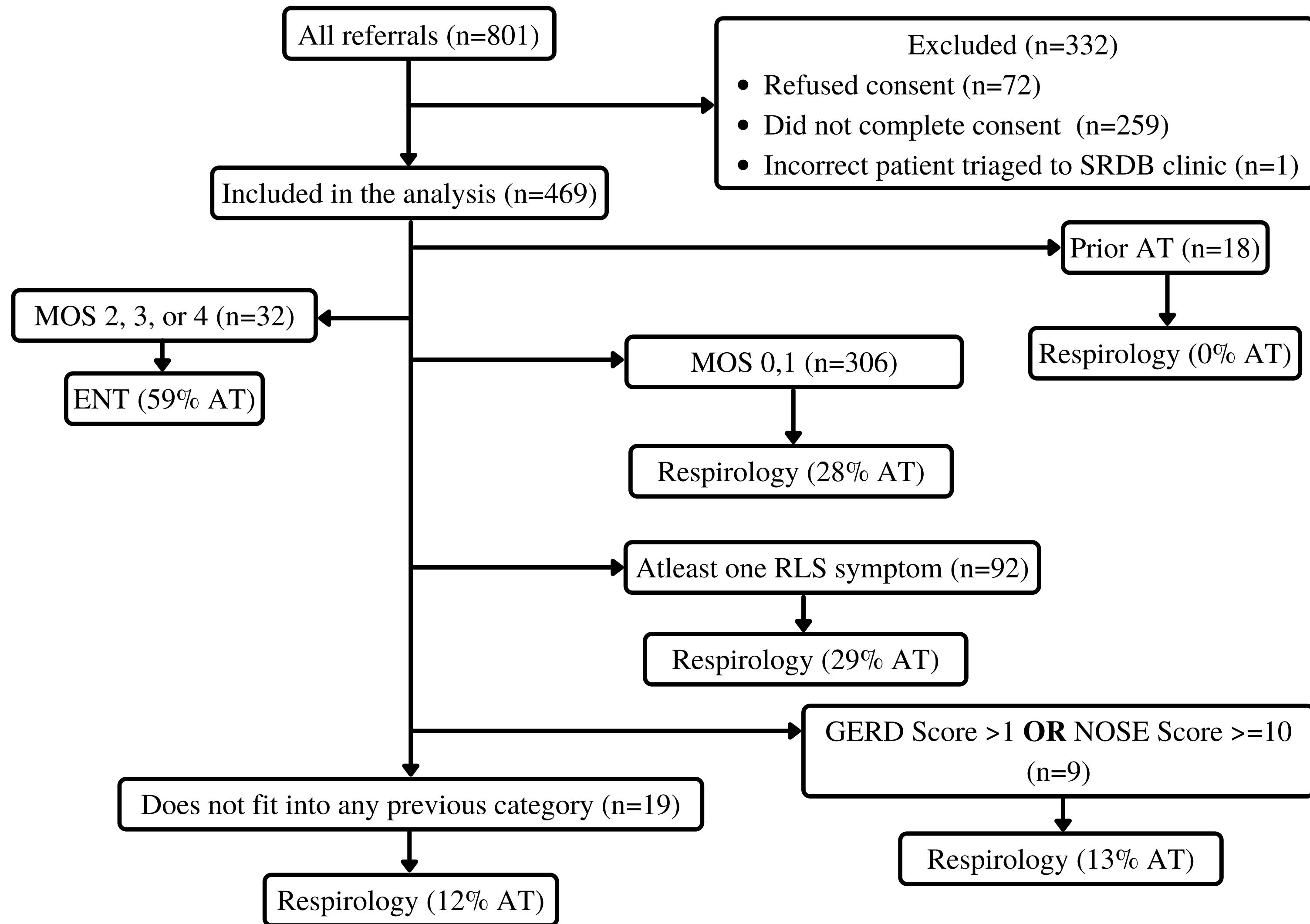

Supplement: Supplementary file 1 — Additional file 1: Contains an additional triage model that uses oximetry data alone. [file 40463_2021_528_MOESM1_ESM.pdf]
